# Primary Pulmonary Malignant Melanoma Found While Evaluating New Onset Cough: A Case Presentation and Literature Review

**DOI:** 10.1155/2019/3867831

**Published:** 2019-04-10

**Authors:** Fernando Figueroa Rodriguez, Ahsan Uddin, Justine Nasr

**Affiliations:** ^1^Department of Internal Medicine, Beaumont Health, Royal Oak, MI, USA; ^2^Department of Internal Medicine-Pediatrics, Beaumont Health, Royal Oak, MI, USA

## Abstract

Malignant melanoma is a nonepithelial neoplasm of melanocytes. It is tremendously rare for this condition to primarily involve the respiratory tract, accounting only for 0.01% of the lung malignancies. It often presents as a solitary nodule provoking mass effect and/or obstructive symptom. It most commonly affects patients 50 years old and older, with no gender predilection. Complete surgical excision is the treatment of choice; nevertheless, chemotherapy or radiation might be necessary depending on tumor location and/or metastasis status. Recently, biochemotherapy and immunotherapy have emerged as promising treatment modalities. We present a case of Primary Pulmonary Malignant Melanoma (PPMM) in a 76-year-old male with no previous personal or family history of cancer who presented with new onset nonproductive cough. We also present an analysis with high yield points summarizing clinical features, diagnostic workup, and management of PPMM. Finally, we post a table summarizing all the cases ever reported in English literature.

## 1. Introduction

There are approximately 41,000 melanoma-related deaths in the world yearly and 16,000 new diagnoses every year; nevertheless there are only 41 cases of PPMM reported in the literature since 1916 [1]. Malignant melanoma of the respiratory tract: this malignancy is easily confused with more conventional neoplasms [2]. Its presentation in the pulmonary system can often be asymptomatic or minimally symptomatic leading to a delay in identification and treatment so that many cases are identified at later stages with 67.5% of cases being metastatic at diagnosis. Prognosis is often quite poor and death within six months is the most common outcome. Our case report seeks to add to the literature for this rare and unusual presentation of melanoma of the pulmonary tract of an otherwise low risk patient.

## 2. Case Presentation

**Figure 1:**
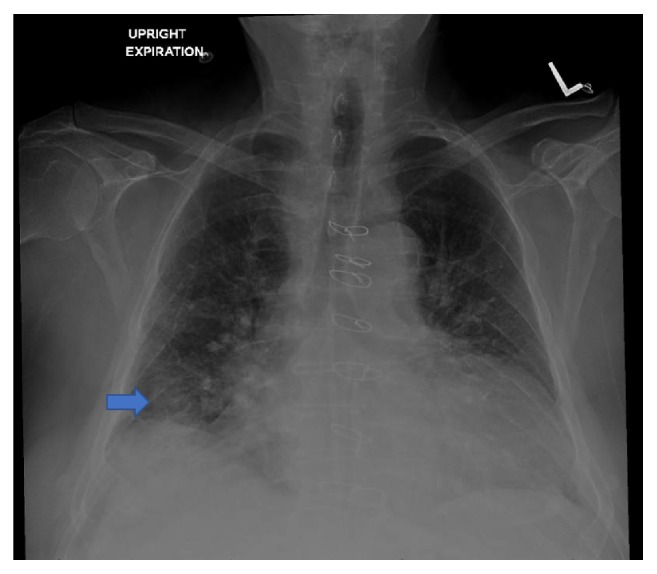
CXR upright-expiration. Arrow: right lower lobe irregular mass like opacity, no evidence of pneumothorax, effusion, or consolidation.

**Figure 2:**
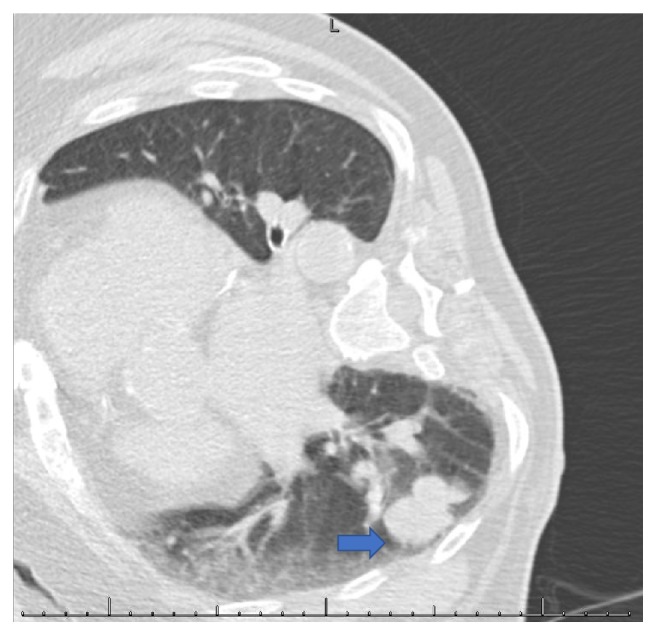
Chest CT. Arrow: 3.2x2x4.3 cm mass in the superior segment of the right lower lobe.

**Figure 3:**
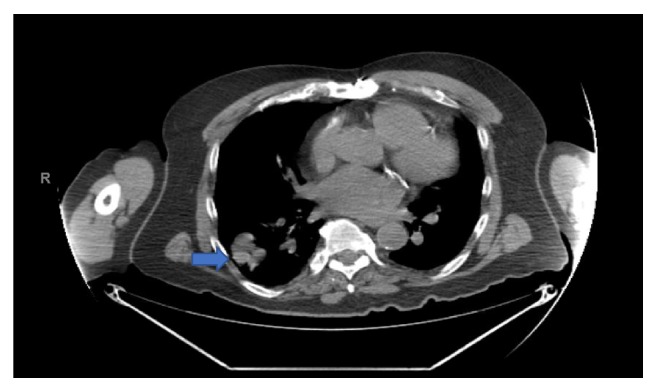
PET scan. Arrow: moderate to intensely increased metabolic activity in the right lower lobe mass (max SUV 8.4 initially increasing to 15.5 on delayed images), without evidence of other focal abnormalities.

**Figure 4:**
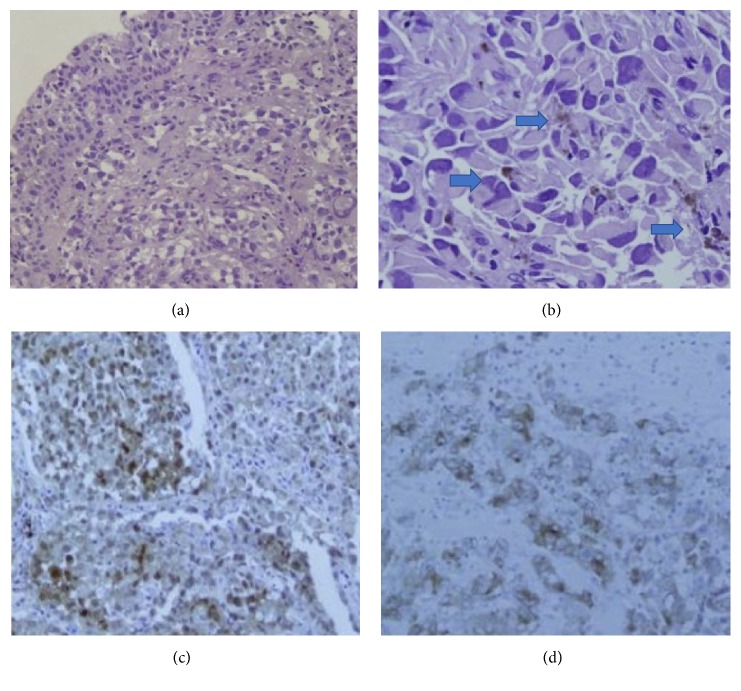
H&E stain ((a) 200x; (b) 400x) with tumor cells located under bronchial mucosa composed of epithelioid tumor cells with large amounts of acid cytoplasm and prominent nuclei. Mitotic figures are easily found. Note dark brown pigment in tumor cells (Arrows). Immunohistochemically, tumor cells were positive for S-100 and HBM 45 ((c) and (d)), amongst others.

A 76-year-old nonsmoker male with history of Obstructive Sleep Apnea presented for elective a Left Knee Total Arthroplasty. Next day, after a successful intervention the patient developed a nonproductive continuous cough. A Chest X-Ray (CXR) was obtained and showed a nodular, irregular opacity in the right lung (Figure 1). Computerized Tomography (CT) of the Chest followed and demonstrated a 3.2x2x4.3 cm mass in the superior segment of the right lower lobe as well as a 1.6 cm subcarinal lymph node (Figure 2). A Positron Emission Tomography (PET) Scan revealed activity in the right lower lobe on both early and delayed imaging (Figure 3). No other focal abnormalities were seen in the rest of the body. A core biopsy of the right lower lobe revealed an invasive, poorly differentiated, malignant melanoma (Box 1 and Figure 4). He was instructed to follow with pulmonary medicine after discharge and two months later, he underwent elective bronchoscopic wedge resection of the right lower lobe with lymph node dissection and biopsies, as well as biopsies of multiple structures in the respiratory tract. Ultimately, he was diagnosed with 3.7 cm malignant melanoma with negative margins and no evidence of metastasis; thus, no chemotherapy or radiation was indicated (Box 2). Serial Repeat CXR and CT scans have shown stable postoperative changes but no signs of recurrence. To date, three years and eight months after diagnosis, the patient continues to follow with his pulmonologist and oncologist every 6 months for surveillance visits; no recurrence has been documented so far.

## 3. Discussion

Malignant melanoma of the skin and mucosae is routinely described as a dark, irregular, and asymmetric lesion, which in its most common form leads to a straight forward diagnosis. In the respiratory tract, this malignancy is easily confused with more conventional neoplasms [2]. Although there are no identified risk factors, the incidence of Primary Pulmonary Malignant Melanoma in nonsmokers is exceptionally rare. To date, there are only two cases described in the literature [1]. Our patient is a lifelong nonsmoker.

Due to its endobronchial location, PPMM frequently manifest with symptoms of cough, hemoptysis, dull chest pain, obstructive symptoms such as pneumonia, lobar collapse, or atelectasis. Symptoms of fatigue, malaise, and nighttime diaphoresis and signs of weight loss or lymphadenopathy might accompany the clinical picture as well. In 20-30% of the cases, it will be incidentally found on imaging [2]. Our patient had nonproductive cough as the only presenting symptom.

Pathogenesis is poorly understood; the most accepted theory involves melanocytes migration along with the primordial tubular respiratory tract during embryogenesis which are also present in the esophagus and pharynx. It has also been proposed that neuroendocrine precursor cells have the potential to undergo melanocytic differentiation [43]; finally, the last theory is spontaneous regression of malignant melanoma, which is defined by the complete disappearance of melanocytic neoplastic cells, that is, a primary dermal lesion that generates disseminated disease and then disappears, leading to misclassifying the neoplasm as primary, when it is truly secondary or metastatic [2]. Unfortunately, none of these theories have been fully accepted.

The definitive diagnosis of PPMM is achieved via clinical, radiological, and pathological findings. There are 6 proposed criteria for diagnosis [43].

1. Junctional changes (nesting) of melanocytes beneath the bronchial epithelium.
2. Invasion of bronchial epithelium by melanoma cells.
3. Obvious melanoma cells by immunohistochemical staining.
4. Solitary lung tumor, with pathology consistent with a primary tumor.
5. Absence of a cutaneous, mucous membrane, or ocular melanoma.
6. Absence of any other detectable tumor at the time of diagnosis.

The main differential diagnoses to consider are melanocytic carcinoid tumor, melanotic paraganglioma, melanotic schwannoma, and pulmonary metastasis of a malignant melanoma. These tumors can often be excluded by a complete history and physical exam but most importantly, by histopathological and immunohistochemical staining, that is, melanoma cells that stain for S-100 and HMB-45, and possibly by electron microscopy) [39].

Treatment of choice is surgical resection with lymph node dissection; the goal is to obtain negative margins (lobectomy or pneumonectomy). Interferon a-2b is frequently offered to mucosal melanoma patients but has not been formally evaluated in this patient population. Similarly, radiation has no clear role [1]. Biochemotherapy which consists of combination of chemotherapy and immunotherapy has been historically an acceptable choice after taking into consideration the tolerability [44].

Vemurafenib was approved by the Food and Drug Administration (FDA) in 2011 as it blocks the serine/threonine kinase BRAF protein which is found in up to 50% of the melanomas [45]. Dabrafenib was approved approval in 2013 [46], which does act against another subtype of protein (BRAF-V600K and E). In the same year, Trametinib, the first Mitogen Activated Protein Kinase inhibitor, was approved and were used in combination with BRAF inhibitors with excellent results (remission within weeks) although resistance to therapy commonly arises after a median of 6 months. Recently immunotherapy with programmed death-1 (PD-1) checkpoint inhibitors like nivolumab and pembrolizumab has been approved by the FDA for the treatment of metastatic cutaneous melanoma [47]. Palliative radiation therapy is utilized when bulky metastatic disease is present. The above medications were developed for mucosal and cutaneous melanoma but may have an increasing role in the management of PPMM

Per current literature review, if detected and treated in early stages (1 and 2) survival rates range from 60 to 85% at 5 years, whereas stages 3 and 4 range from 15 to 25% only. Late recurrence is uncommon with a reported incidence of 2.4%. Our patient was determined to be at stage 1 at the time of diagnosis; he has survived for almost 4 years and continues to be in remission [48].

## 4. Literature Review

There are approximately 41,000 melanoma-related deaths in the world yearly and 16,000 new diagnoses every year; nevertheless there are only 41 cases of PPMM reported in the literature since 1916 (see Table 1) [1]. Prognosis is often quite poor and death within six months is the most common outcome.

Given the broad period in which these cases were published, as well as new advances in diagnostic and therapeutic technology, conclusions regarding treatment modalities or the variables that impact survival and mortality are precluded. There are only a few facts that can be stated upon review of the cases above:

- Gender distribution was fairly equal with 52% of the patients being males and 48% females.
- The median age at time of diagnosis was 59.1 years (29 to 90).
- The average tumor size was 4.6 cm (1.0 to 10 cm).
- 67.5% of the patients had metastasis at the time of diagnosis. The most common sites were contralateral lung, liver, brain, and bones as detailed above.

## 5. Conclusion

With this case report and literature review we aim to add critical data to the current medical literature regarding this highly unusual and rare disease. Reliable data regarding clinical presentation, diagnosis, and potential treatments of PPMM is becoming more available to health care workers making this a promising era in the identification and management of this condition, specifically with the establishment of unequivocal diagnostic criteria and the advent of new treatment modalities. Additionally, our findings of disseminated disease and prompt surgical intervention being the strongest factors influencing survival are perfectly exemplified by our case report. At this time randomized studies are univariably defiant as with any rare disorder.

## Figures and Tables

**Box 1:**
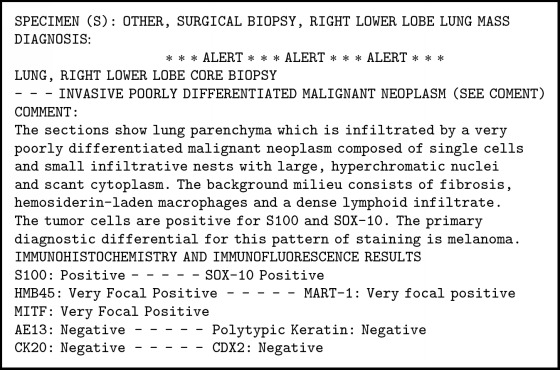
Biopsy report.

**Box 2:**
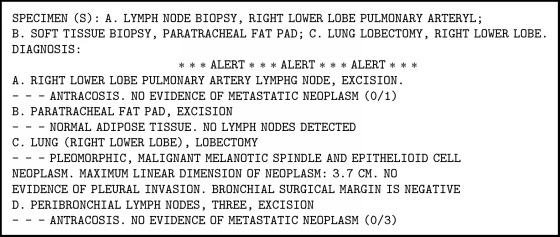
Bronchoscopy and second biopsy report.

**Table 1:** 

Case	Age, Gender	Tumor Size (cm)	Surgery	Adjuvant	Metastasis	Outcome
1 [3]	40 F	2.0	None	None	Not Reported	Died 8 months after diagnosis
2 [4]	48 F	5.0	Pneumonectomy	None	Not Reported	Died after surgery
3 [5]	45 M	2.0	Pneumonectomy	None	Contralateral Lung	Died 6 months after diagnosis
4 [6]	71 M	3.5	Lobectomy	None	None	Alive after 10 years
5 [7]	60 F	4.5	Pneumonectomy	None	None	Alive after 11 years
6 [8]	61 F	10	Segmental Resection	None	Contralateral Lung, Brain	Died 7 months after surgery
7 [9]	40 F	5.0	Lobectomy	None	Not Reported	Not Reported
8 [10]	56 M	4.0	Lobectomy	None	Contralateral Lung, Brain, Liver	Died 14 months after surgery
9 [11]	40 M	2.5	Pneumonectomy	None	None	Alive after 3 years
10 [12]	62 F	Not Reported	Thoracotomy	Radiation	Liver, Eye	Died 4 months after surgery
11 [13]	70 F	Not Reported	None	Radiation	Liver, Ribs, Lymph Nodes	Died 9 weeks after diagnosis
12 [14]	47 M	Not Reported	None	None	None	Died shortly after diagnosis
13 [15]	29 F	5.0	Lobectomy	Chemotherapy	Contralateral Lung, Liver, Heart, Bone	Died 1 month after surgery
14 [16]	80 M	1.5	Excisional Biopsy	Radiation	Contralateral Lung	Died 5.5 months after diagnosis
15 [17]	56 M	4.0	Pneumonectomy	Chemotherapy	Contralateral Lung, Heart	Died 1 month after surgery
16 [18]	42 F	6.0	Lobectomy	None	None	Alive 2.5 year after surgery
17 [19]	58 M	5.0	Lobectomy	None	None	Alive 18 months after surgery
18 [20]	62 M	1.0	Lobectomy	None	Heart, Lymph Nodes	Died 2 months after surgery
19 [21]	30 F	3.0	Lobectomy	None	Contralateral Lung, Heart, Brain	Died 5 months after surgery
20 [22]	90 M	6.0	None	None	Not Reported	Died at the time of diagnosis
21 [23]	59 M	8.0	Lobectomy	Alfa-Interferon	None	Alive 30 months after surgery
22 [24]	41 F	Not Reported	Pneumonectomy	Alfa-Interferon	None	Alive 18 months after surgery
23 [25]	74 M	8.0	Lobectomy	Chemotherapy	None	Died 10 months after surgery
24 [26]	68 F	5.0	Lobectomy	Alfa-Interferon	None	Alive 6 years after surgery
25 [27]	89 M	4.5	Lobectomy	None	None	Alive 5 years after surgery
26 [28]	58 M	2.8	Lobectomy	Dacarbazine	Contralateral Lung	Died 6 months after surgery
27 [29]	69 F	4.0	Lobectomy	Darbazine	Contralateral Lung, Liver, Brain, Skin	Died 6 months after surgery
28 [30]	52 F	2.0 - 2.4	None	Dacarbazine	Brain	Died 4 months after surgery
29 [31]	65 F	6.0	None	Darbazine	None	Alive 6 months after surgery
30 [32]	68 M	5.0	Pneumonectomy	Dacarbazine, Vincristine, Nimustine	Contralateral Lung	Died 2 months after diagnosis
31 [33]	62 F	5.0	None	Dacarbazine	Thoracic Vertebrae, Pleura	Alive 12 months after diagnosis
32 [34]	56 M	3.2	Pneumonectomy	Radiation	Cervical Spine	Alive 6 months after diagnosis
33 [35]	60 M	4.5	Pneumonectomy	Alfa-Interferon	None	Alive 18 months aftr surgery
34 [36]	58 F	9.0	None	None	None	Died 2 months after diagnosis
35 [37]	69 F	6.0	Lobectomy	Dacarbazine	None	Died 6 months after surgery
36 [38]	63 M	5.0	None	None	None	Died 2 months after diagnosis
37 [39]	82 F	8.0	None	None	None	Died 3 months after diagnosis
38 [40]	55 M	3.0	None	Dacarbazine, Alfa-Interferon	Skin, Brain	Died 3 months after diagnosis
39 [41]	69 M	5.0	None	None	Brain, Thoracic Vertebrae	Died 3 months after diagnosis
40 [42]	60 M	3.0	None	None	Lymph Nodes	Died 2 months after diagnosis
41 [1]	56 F	Not Reported	None	Dacarbazine, Alfa – Interferon, Ipilimumab	Brain	Died 5 months after diagnosis

## References

[1] Kyriakopoulos C., Zarkavelis G. (2017). Primary pulmonary malignant melanoma: report of an important entity and literature review. *Case Reports in Oncological Medicine*.

[2] Khosravi H., Akabane A. L., Alloo A., Nazarian R. M., Boland G. M. (2016). Metastatic melanoma with spontaneous complete regression of a thick primary lesion. *JAAD Case Reports*.

[3] Kunkel O. F., Torrey E. (1916). Report of a case of primary melanotic sarcoma of lung presenting difficulties in differentiating from tuberculosis. *New York State Journal of Medicine*.

[4] Carlucci G. A., Schleussner R. C. (1941-1942). Primary (?) melanoma of the lung: a case report. *The Journal of Thoracic and Cardiovascular Surgery*.

[5] Salm R. (1963). A primary malignant melanoma of the bronchus. *The Journal of Pathology*.

[6] Reed R. J., Kent E. M. (1964). Solitary pulmonary melanomas: two case reports. *The Journal of Thoracic and Cardiovascular Surgery*.

[7] Reid J. D., Mehta V. T. (1966). Melanoma of the lower respiratory tract. *Cancer*.

[8] Jensen O. A., Egedorf J. (1967). Primary malignant melanoma of the lung. *Scandinavian journal of respiratory diseases*.

[9] Allen M. S., Drash E. C. (1968). Primary melanoma of the lung. *Cancer*.

[10] Taboada C. F., McMurray J. D., Jordan R. A., Seybold W. D. (1972). Primary melanoma of the lung. *Chest*.

[11] Weshler Z., Sulkes A., Kopolovitch J., Leviatan A., Shifrin E. (1980). Bronchial malignant melanoma. *Journal of Surgical Oncology*.

[12] Robertson A. J., Sinclair D. J. M., Sutton P. P., Guthrie W. (1980). Primary melanocarcinoma of the lower respiratory tract. *Thorax*.

[13] Gephardt G. N. (1981). Malignant melanoma of the bronchus. *Human Pathology*.

[14] Carstens P. H. B., Kuhns J. G., Ghazi C. (1984). Primary malignant melanomas of the lung and adrenal. *Human Pathology*.

[15] Cagle P., Mace M. L., Judge D. M., Teague R. B., Wilson R. K., Greenberg S. D. (1984). Pulmonary melanoma. Primary vs metastatic. *CHEST*.

[16] Demeter S. L., Fuenning C., Miller J. B. (1987). Primary malignant melanoma of the lower respiratory tract: endoscopic identification. *Cleveland Clinic Journal of Medicine*.

[17] Alghanem A. A., Mehan J., Hassan A. A. (1987). Primary malignant melanoma of the lung. *Journal of Surgical Oncology*.

[18] Santos F., Entrenas L. M., Sebastian F. (1987). Primary bronchopulmonary malignant melanoma: case report. *Scandinavian Cardiovascular Journal*.

[19] Bagwell S. P., Flynn S. D., Cox P. M., Davison J. A. (1989). Primary malignant melanoma of the lung. *American Review of Respiratory Disease*.

[20] Bertola G., Pasquotti B., Morassut S., Massarut S., Sigon R., Rossi C. (1989). Primary lung melanoma. *Italian Journal of Surgical Sciences*.

[21] Ost D., Joseph C., Sogoloff H., Menezes G. (1999). Primary pulmonary melanoma: case report and literature review. *Mayo Clinic Proceedings*.

[22] Erdal N. B., Karakurt Z., Pandul I., Tahaoğlu C. (2000). A case report: primary pulmonary melanoma. *Turkish Respiratory Journal*.

[23] Dountsis A., Zisis C., Karagianni E., Dahabreh J. (2003). Primary malignant melanoma of the lung: a case report. *World Journal of Surgical Oncology*.

[24] Reddy V. S., Mykytenko J., Giltman L. I., Mansour K. A. (2007). Primary malignant melanoma of the lung: review of literature and report of a case. *The American Surgeon*.

[25] Zuckermann B., Papiashvilli M., Bar I. (2011). Primary pulmonary malignant melanoma of right upper lobe of lung. * Israel Medical Association Journal*.

[26] Seitelman E., Donenfeld P., Kay K., Takabe K., Andaz S., Fox S. (2011). Successful treatment of primary pulmonary melanoma. *Journal of Thoracic Disease*.

[27] Neri S., Komatsu T., Kitamura J., Otsuka K., Katakami N., Takahashi Y. (2011). Malignant melanoma of the lung: report of two cases. *Annals of Thoracic and Cardiovascular Surgery*.

[28] Gong L., Liu X., Zhang W. (2012). Primary pulmonary malignant melanoma: a clinicopathologic study of two cases. *Diagnostic Pathology*.

[29] Ouarssani A., Atoini F., Reda R., Lhou F. A., Rguibi M. I. (2012). Malignant melanoma of the lung: a case report. *Pan African Medical Journal*.

[30] Dos Santos C. L., Fernandes L. R., Meruje M., Barata F. (2013). Primary pulmonary melanoma: the unexpected tumor. *BMJ Case Reports*.

[31] Kamaleshwaran K., Natarajan S., Parthiban J., Mehta S., Radhakrishnan K., Shinto A. (2014). Rare case of extradural spinal metastasis from primary lung malignant melanoma detected with fluorine-18 fluorodeoxyglucose-positron emission tomography/computed tomography. *Indian Journal of Nuclear Medicine*.

[32] Zhang X., Wang Y., Du J. (2015). Primary malignant melanoma of left lower lobe of lung: a case report and review of the literature. *Oncology Letters*.

[33] Gupta A., Bhattacharya D., Jain S., Suri J. C. (2015). Primary malignant melanoma of the lung: case report and literature review. *The Indian Journal of Chest Diseases & Allied Sciences*.

[34] Postrzech-Adamczyk K., Chabowski M., Głuszczyk-Ferenc B. (2015). Malignant melanoma of the lung: case series. *Kardiochirurgia i Torakochirurgia Polska*.

[35] Hwang K., Hwang K., Jung J. (2015). Primary pulmonary malignant melanoma: an unexpected tumor. *Tuberculosis and Respiratory Diseases*.

[36] Filippini A., Zorzi F., Bna' C., Arnaboldi A., Sabatini T. (2015). Dark sputum: an atypical presentation of primary pulmonary malignant melanoma. *Respiratory Medicine Case Reports*.

[37] Kim S. R., Yoon H.-Y., Jin G. Y., Choe Y. H., Park S. Y., Lee Y. C. (2016). Pulmonary malignant melanoma with distant metastasis assessed by positron emission tomography-computed tomography. *Thoracic Cancer*.

[38] Feng Y., Zhao J., Yang Q. (2016). Pulmonary melanoma and “crazy paving” patterns in chest images: a case report and literature review. *BMC Cancer*.

[39] Maeda R., Isowa N., Onuma H., Miura H., Tokuyasu H., Kawasaki Y. (2009). Primary malignant melanoma of the lung with rapid progression. *General Thoracic and Cardiovascular Surgery*.

[40] Singh K., Sharma M. C., Jain D., Kumar R. (2008). Melanotic medullary carcinoma of thyroid—report of a rare case with brief review of literature. *Diagnostic Pathology*.

[41] Iihara K., Yamaguchi K., Fujioka Y., Uno S. (2002). Pigmented neuroendocrine tumor of the lung, showing neuromelanin. *Pathology International*.

[42] Petersen R. P., Hanish S. I., Haney J. C. (2007). Improved survival with pulmonary metastasectomy: an analysis of 1720 patients with pulmonary metastatic melanoma. *The Journal of Thoracic and Cardiovascular Surgery*.

[43] Apostolos D., Zisis C. (2003). Primary malignant melanoma of the lung: a case report. *World Journal of Surgical Oncology*.

[44] Dummer R., Hauschild A., Lindenblatt N., Pentheroudakis G., Keilholz U. (2015). Cutaneous melanoma: ESMO clinical practice guidelines for diagnosis, treatment and follow-up. *Annals of Oncology*.

[45] Long G. V., Menzies A. M., Nagrial A. M. (2011). Prognostic and clinicopathologic associations of oncogenic BRAF in metastatic melanoma. *Journal of Clinical Oncology*.

[46] Ascierto P. A., Minor D., Ribas A. (2013). Phase II trial (BREAK-2) of the BRAF inhibitor dabrafenib (GSK2118436) in patients with metastatic melanoma. *Journal of Clinical Oncology*.

[47] Yun S., Vincelette N. D., Green M. R., Wahner Hendrickson A. E., Abraham I. (2016). Targeting immune checkpoints in unresectable metastatic cutaneous melanoma: a systematic review and meta-analysis of anti-CTLA-4 and anti-PD-1 agents trials. *Cancer Medicine*.

[48] Ugurel S., Röhmel J., Ascierto P. A. (2016). Survival of patients with advanced metastatic melanoma: the impact of novel therapies. *European Journal of Cancer*.

